# Observational study of the effect of metaphylaxis treatment on feedlot cattle productivity and health

**DOI:** 10.3389/fvets.2022.947585

**Published:** 2022-07-22

**Authors:** William E. Maples, B. Wade Brorsen, Derrell Peel, Britt Hicks

**Affiliations:** ^1^Department of Agricultural Economics, Mississippi State University, Starkville, MS, United States; ^2^Department of Agricultural Economics, Oklahoma State University, Stillwater, OK, United States; ^3^Oklahoma Cooperative Extension Service, Oklahoma Panhandle Research and Extension Center, Goodwell, OK, United States

**Keywords:** antibiotics, economics of metaphylaxis, antimicrobial, propensity score matching, bovine respiratory disease

## Abstract

There is public pressure to reduce antimicrobial use in livestock production. Metaphylaxis usage raises special concern as it is given to a whole group of animals. The objective of this research was to determine the difference in cattle productivity and health (average daily gain, death loss, etc.) between cattle given metaphylaxis and those to which it was not given. Observational data were provided by a commercial feedlot in the Southern Great Plains region of the U.S.A. with an operating capacity >50,000 head. Cattle that received metaphylaxis treatment had substantially poorer health outcomes than those that did not. Cattle were more likely to have been given metaphylaxis treatment if they had a lower weight, were from a sale barn, or had been shipped long distances. Propensity score matching was used in an attempt to estimate the effect of metaphylaxis treatment on feedlot cattle. Propensity score matching was unable to overcome the endogeneity issues present in the data (endogeneity results from the animals being more likely to benefit from the treatment being the ones who received it). The dataset had information on cattle weight, state of purchase, and whether or not the cattle were from a sale barn, and so the feedlot must have based the treatment decision on information that was not recorded and therefore not included in the dataset. As an observational study, there are limitations in addition to data limitations, such as the possibility that the feedlot studied might not be representative of others. Even though the effect of metaphylaxis was not identified, the fact that it was unidentifiable supports the argument that the feedlot did treat the animals most likely to need metaphylaxis treatment. This should temper some fear of metaphylaxis treatment being overused and of antimicrobials being given needlessly.

## Introduction

The majority of feedlot deaths are due to bovine respiratory disease (BRD) (1). BRD is the costliest illness in U.S. feedlots (2). BRD is a disease complex resulting from a variety of pathogens and environmental factors. The economic losses from BRD occur due to morbidity, mortality, treatment and prevention costs, loss of production, and reduced carcass value (3).

One strategy that feedlots use to combat BRD in cattle is to administer an antimicrobial. Sometimes, feedlots treat only animals that show signs of clinical illness. Detection of BRD, however, can be a challenge in large pens of cattle because relying solely on visual signs has a high diagnosis error (4). In discussions, the feedlot owners that provided the data for this study cited the difficulty in finding experienced labor that can accurately identify illness when riding the pens. To account for the difficulty in identifying BRD illness, metaphylaxis treatment is an option.

Metaphylaxis is the mass medication of an entire animal population to reduce the incidence of disease in a population that already has some evidence of disease (2, 5, 6). In a feedlot, this usually means the mass medication of a lot of cattle with an antimicrobial on arrival at the feedlot or within the first few days of arrival. Metaphylaxis treatment can reduce the occurrence of BRD and death in highly stressed and newly received cattle (2). Metaphylaxis is a treatment for animals within the population that may be suffering from BRD and also serves to reduce the spread to other animals in the population that are at risk of developing BRD (6).

Metaphylaxis treatment is commonly reserved for cattle at high risk of developing BRD. On arrival at a feedlot, calves should be given a risk score that relates to the probability of developing BRD (7). Since BRD is difficult to diagnose, feedlot managers depend on predisposing factors to determine the risk of developing BRD. Shipping distance from the origin of purchase to the feedlot is one of the most widely accepted predisposing factors for calves to develop BRD (2, 5). This practice is related to an early name for BRD which was commonly referred to as “shipping fever.” During transportation, stress is created by loading and unloading, food and water deprivation, weather conditions at the time of transportation, and standing over long distances.

Another important predisposing factor is the weight of cattle on arrival. Younger, lightweight calves that were recently weaned are more susceptible to BRD. Commingling a lot of cattle from multiple sources increases the risk of BRD due to stress and more exposure to potential pathogens (8). Cattle from auction markets are likewise at a higher risk (8). Other predisposing factors include the previous health history and sex of the animal. Feedlot managers must still use their own experience along with these known risk factors when estimating the risk of BRD (6).

Numerous experimental studies have measured the impact of metaphylaxis treatment on cattle health and performance. Using metaphylaxis treatment on cattle at a high risk of developing BRD has consistently been shown to decrease morbidity and mortality (5). Abell et al. conducted a meta-analysis of 37 field trials of metaphylaxis effects on BRD (9). They indicated that metaphylaxis of cattle on arrival at the feedlot using upper tier treatments reduced morbidity by 80–90%. However, there were insufficient data to determine the effects of mass medication on mortality. The meta-analysis by Wileman concluded that metaphylaxis treatment given on arrival to feeder cattle increased average daily gain by 0.24 pounds/d (0.11kg/d) compared to animals that did not receive treatment (10). Wileman also found that mortality was 1.8% for animals receiving metaphylaxis compared to 3.8% for untreated animals.

Some consumers prefer meat from animals not treated with antibiotics or antimicrobials (11). As consumer preference has shifted toward antibiotic-free production, various large restaurant chains and supermarkets are responding by committing to reduce or completely ban antibiotic use in meat sold (12). Much of the antibiotic reduction to date has focused on chicken, for example, in 2016, McDonald's quit serving meat from chickens that had been treated with antibiotics.

Much of the concern with antimicrobial use in meat production is related to the development of antimicrobial-resistant pathogens in humans (13). The connection between antimicrobial use in livestock and the relationship to antimicrobial-resistant pathogens in humans is still being heavily researched and debated. The use of antimicrobials in livestock production and a possible link to human health risk and antimicrobial resistance is a concern for both federal and international agencies (14, 15). In feedlots, antimicrobials have been used for growth promotion, disease prevention and control, and disease treatment. The Food and Drug Administration with “Guidance for Industry #213” began curtailing the use of medically important antimicrobials for growth promotion in livestock (16, 17). There are no current plans to limit the use of antimicrobials in metaphylaxis since it is associated with disease control.

In the current environment of concerns about unnecessary antimicrobial use in feedlots, it is reasonable to ask what the effect of a metaphylaxis ban would be on feedlot producers. A recent paper by Dennis et al. estimated the value of metaphylaxis treatment for the U.S. cattle industry (18). The authors estimated the direct net return value of metaphylaxis to the U.S. fed cattle industry to be at least $532 million and found beef producer surplus losses of $1.8 billion if metaphylaxis was eliminated. The authors accomplished their objective using simulation techniques and assuming mortality distributions across cattle placement weight categories and metaphylaxis using categories of no metaphylaxis or metaphylaxis. Dennis et al. clearly show that a ban on metaphylaxis treatment will have a serious impact on the U.S. beef industry (18).

The objective of this paper was to determine the effect of metaphylaxis treatment on animal health and growth. The goal is similar to Dennis et al., but uses different data, uses some different methods, and provides some information not provided in Dennis et al. (18). Observational data were used to demonstrate how antimicrobials were used in a real feedlot situation. An issue of concern is that the cattle that were given metaphylaxis are different than those that were not given. The decision to use metaphylaxis is not random and is greater for lots that are more likely to develop BRD. Propensity score matching attempts to correct the bias created by the feedlot treating the cattle that were the most likely to get sick. Propensity score matching attempts to match each treated lot with a lot that has similar characteristics. Cattle here were matched based on initial weight, state of origin, and whether they are purchased at a sale barn. Since propensity score matching failed to correct for the bias, it implies that the feedlot used information about the cattle beyond the variables included in the model (such as whether or not the cattle were preconditioned). As an alternative approach, the data here are then combined with the methods used by Dennis et al. to create death loss distributions for animals that received metaphylaxis and death loss distributions for the animals that did not receive metaphylaxis.

## Materials and methods

### Data

A large commercial feeding operation in the Southern Plains of the United States provided data from a continuous 7-year period (2009–2016). The feedlot provided adequate animal husbandry, and no animal interventions beyond industry-accepted diagnostic and therapeutic practices were used. The observational data included closeout and health information for individual lots of cattle at the feedlot. Data were provided in raw Microsoft Access files as entered by feedlot staff with all data sorting and aggregation done by the authors. While the feedlot did not specifically record if a lot received metaphylaxis, they did provide dates of all medication received. The authors used the provided medication data to separate lots that received metaphylaxis on arrival and lots that received no metaphylaxis. The no metaphylaxis lots received no antimicrobials during processing at arrival. The metaphylaxis lots were administered antimicrobials using a metaphylaxis treatment at arrival. This group consists of lots in which 100% of the animals received antimicrobials on arrival. Since metaphylaxis treatment is given to the entire pen, the unit of observation is the lot. Only death loss and medical treatment were available for individual animals, and so the data for these variables are pen averages.

In total, the feedlot provided information on 4,970 lots of cattle. Of the total data provided, 437 lots were excluded from the analysis due to the inability to place the lot into the metaphylaxis or no metaphylaxis groups. Medication data on the excluded lots indicated that only a portion of the lot received antimicrobials on arrival, which did not fit the authors definition of metaphylaxis. In total, the data analyzed for this study include 4,533 lots of cattle. There are 1,188 lots that received metaphylaxis treatment and 3,345 lots that did not receive metaphylaxis. The feedlot that provided the data either uses metaphylaxis on arrival or does not use metaphylaxis. In discussion with the feedlot owners, they stated they avoid attempting to diagnose sick animals at arrival due to the difficulty of identification.

Percent hospital head days represent the number of head days a lot spent in the hospital pen as a percent of total head days. Total death loss is the percent of animals that died, and respiratory death loss is the percent of animals in a lot that died from a respiratory illness. Only deaths in the first 45 days of feeding are included, which is the conventional time period to consider the effects of BRD (19, 20). Medicine cost per head is total cost of medication after processing. This cost does not include any vaccines or antimicrobials the animal received upon arriving at the feedlot. Processing cost per head includes the costs of antimicrobials, vaccines, implants, etc. that an animal receives when they arrive at the feedlot.

### Estimation methods

An issue of concern is that the cattle that make up the different groups are distinctly different. The decision to use metaphylaxis during the processing phase is not random but is based on the chance that the lot will develop BRD. This introduces a selectivity bias that must be accounted for when estimating the treatment effect.

Propensity score matching is used to estimate the treatment effects of metaphylaxis use. The treatment effect framework attempts to infer a causal connection between a treatment and outcome. Since there are more no metaphylaxis treated lots in the data, they will be considered the control group, *D* = 0. The treatment group, *D* = 1, will refer to lots that were medicated using metaphylaxis. Following the framework of Abadie et al., for lot *i*, *i* = 1, …, *N*, let {*Y*_*i*_(0), *Y*_*i*_(1)} denote the two potential outcomes: *Y*_*i*_(0) is the outcome of lot *i* when receiving no metaphylaxis treatment and *Y*_*i*_(1) is the outcome of lot *i* when animals are treated with metaphylaxis.

If both *Y*_*i*_(0) and *Y*_*i*_(1) are observable, then the effect of using metaphylaxis on lot *i* instead of no metaphylaxis would be directly observable as *Y*_*i*_(1)−*Y*_*i*_(0). The average treatment effect (ATE), α_*ATE*_, can then be calculated as follows:


(1) $$\begin{array}{l} \alpha_{ATE}=E\left\{Y\left(1\right)-Y\left(0\right)\right\}. \end{array}$$

The ATE is interpreted as the expected gain or loss that a randomly selected individual receives from participating in the treatment. In the case of this paper, ATE is the expected gain or loss that a randomly selected lot of cattle would experience if administered metaphylaxis.

The average treatment effects for the subpopulation group of treated lots can also be defined. This effect is referred to as the average treatment effect on the treated (ATT), α_*ATT*_, and is defined as follows:


(2) $$\begin{array}{l} \alpha_{ATT}=E\left\{Y\left(1\right)-Y\left(0\right)|D=1\right\}. \end{array}$$

The ATT is interpreted as the average gain or loss from the treatment for individuals receiving the treatment. The interpretation is that for lots treated with metaphylaxis, it is the average gain or loss in a production or health outcome due to being treated with metaphylaxis instead of no metaphylaxis.

The average treatment effect on the control (ATC), α_*ATC*_, can likewise be defined as follows:


(3) $$\begin{array}{l} \alpha_{ATC}=E\left\{Y\left(1\right)-Y\left(0\right)|D=0\right\} \end{array}$$

The ATC is interpreted as the effect for non-participants if they had participated in the treatment. For this paper, the interpretation is the average gain or loss in a production or health outcome that lots who received no metaphylaxis would have seen if they had instead been treated using metaphylaxis.

In Equations 1–3, only one of the two outcomes is observed. An animal cannot be in both the treated and control groups. Let the observed outcome be denoted by *Y*_*i*_:


(4) $$\begin{array}{l} Y_{i}=Y_{i}\left(D_{i}\right)=[\begin{array}{c} Y_{i}(0)\\ Y_{i}(1) \end{array}\;\begin{array}{c} if\;D_{i}=0\\ if\;D_{i}=1 \end{array} \end{array}$$

Estimating the average treatment effect requires estimating the unobserved potential outcome for each observation in the sample. The estimation can be accomplished by matching. The basic idea behind matching is that if the decision to treat is “random” for individuals with similar pretreatment variables, the outcomes of similar individuals who were not treated can be used to estimate the untreated outcome. The matching of similar individuals leads to matching estimators.

The most basic matching estimator is the simple matching estimator, α_*sm*_. Let *Ŷ*_*i*_(1) and *Ŷ*_*i*_(0) represent the unobserved matched outcome for lot *i*. The simple matching estimator for the ATE is specified as follows:


(5) $$\begin{array}{l} \alpha_{sm.ATE}=\frac{1}{N}\sum_{i=1}^{N}\left\{{\hat{Y}}_{i}(1)-{\hat{Y}}_{i}(0)\right\}. \end{array}$$

The ATE estimator can be modified to estimate the ATT and ATC. These estimators are specified as follows:


(6) $$\begin{array}{l} \alpha_{sm.ATT}\;=\frac{1}{N_{1}}\sum_{i:D=1}\left\{Y_{i}-{\hat{Y}}_{i}(0)\right\} \end{array}$$

(7) $$\begin{array}{l} \alpha_{sm.ATC}\;=\frac{1}{N_{0}}\sum_{i:D=0}\left\{{\hat{Y}}_{i}\left(1\right)-Y_{i}\right\} \end{array}$$

where *N*_0_ and *N*_1_ represent the number of observations in the control group and treatment group, respectively.

As stated above, matching is done on a set of pretreatment variables, which are represented as X. An important assumption in treatment effects estimation is the conditional independence assumption (CIA) that states that conditional on X, the outcomes are independent of treatment (21). When this assumption is presumed to hold, it implies that treatment status is random conditional on the vector of observable variables, X. Matching is therefore a quasi-experimental technique, which attempts to replicate actual experimental conditions. In an observational study, the CIA means that treatment can be said to be “as good as randomly assigned,” conditional on the vector of observed variables (22).

Propensity score estimation allows matching observations conditional on X. A propensity score is the conditional probability of receiving treatment given X, denoted as *p*(**X**), and was first suggested by Rosenbaum and Rubin (23). If matching on X is justified, then matching on *p*(**X**) is justified as well (24). The propensity score is estimated here using a logistic regression:


(8) $$\begin{array}{l} P\left(D_{i}=1|{\text{X}}_{\text{i}}\right)=\frac{e^{F\left({\text{X}}_{\text{i}}\right)}}{1-e^{F\left({\text{X}}_{\text{i}}\right)}} \end{array}$$

where *P*(*D*_*i*_ = 1|**x**_**i**_) is the probability that *D*_*i*_ equals one given **X**_**i**_ and *F*(**X**_**i**_) are a function of explanatory variables. The explanatory variables include the weight of cattle upon arrival at the feedlot, the state of origin of the lot, the sex of the lot, whether that lot is purchased from a sale barn, and the season of the year the lot was placed. The propensity score in this paper represents the risk score of a lot of cattle.

Once a propensity score is calculated for each lot, it is matched to a similar lot of the opposite treatment using the nearest neighbor matching. The matched lot of the opposite treatment forms the counterfactual for the original lot. Metaphylaxis lots are matched to a lot that received no metaphylaxis, and these no metaphylaxis lots become the counterfactual, Ŷ_*i*_(0). Since there are less metaphylaxis than no metaphylaxis lots, this will be a 1–1 match. Lots that originally received no metaphylaxis are matched to lots from the metaphylaxis group. These metaphylaxis lots create the counterfactual, Ŷ_*i*_(1). This will create an uneven match due to more no metaphylaxis lots.

ATE, ATT, and ATC are estimated for average daily gain, respiratory death loss, death loss in the first 45 days, total death loss, percent of sick head days, and average medicine cost per head post-processing. The treatment effects are estimated in STATA using the PSMATCH2 package (19). Confidence intervals are calculated using the work of Abadie et al. (25).

## Results

Table 1 contains descriptive statistics of the different treatment groups. The two groups are significantly different (*P* < 0.001) for all characteristics considered. The no antimicrobials group has the largest average placement weights of 732 pounds (332 kg), and the metaphylaxis group has an average placement weight of 609 pounds (276 kg). From the descriptive statistics in Table 1, the no metaphylaxis group is on average healthier than the metaphylaxis groups. The no metaphylaxis group has a lower percent of hospital head days, lower total death loss, lower respiratory death loss, and a lower cost of medicine per head after processing. These differences illustrate the bias that is created in estimating the effect of metaphylaxis treatment by simply comparing the two groups.

**Table 1:** Descriptive statistics of the lots of fed cattle included in the study.

**Variable**	**All Lots**	**No Antimicrobials on Arrival**	**Metaphy laxis**	***P*** **-value**
# of lots	4533	3345	1,188	
# head in lot	113	120	91	<0.001
Placement weight	700	732	609	<0.001
Sale weight	1308	1,332	1,241	<0.001
Average daily gain	3.39	3.52	3.04	<0.001
% hospital head days	0.74	0.60	1.13	<0.001
% death loss	2.23	1.65	3.87	<0.001
% respiratory death loss	1.58	1.07	3.02	<0.001
% deaths in 45 days	0.96	0.61	1.94	<0.001
Medicine expenses/$ per head	3.66	3.28	4.74	<0.001
Processing expenses/$ per lot	14.65	9.61	28.82	<0.001

*The P-values are associated with the null hypothesis of no difference in means between those given metaphylaxis and those not given it*.

### Antimicrobial cost and use

Table 1 contains per head average cost of processing a lot of cattle upon arrival. The processing costs include implants, vaccines, and other veterinarian services needed when an animal arrives at the feedlot. Processing is also the stage where metaphylaxis would be administered. The no metaphylaxis group has a per head processing cost of $9.61, and the metaphylaxis group has a per head processing cost of $28.82. Since every animal enters the chute for treatment, the difference in labor cost between the two treatments is assumed to be immaterial. The difference in processing cost of $19.21 (calculated as $28.82 minus $9.61) represents the cost of metaphylaxis treatment. Dennis et al. used an estimated cost of metaphylaxis treatment of $23.81 (18).

We can further break down metaphylaxis treatment by the type of antimicrobial. Five main antimicrobials used for metaphylaxis treatment that appear in the dataset are Draxxin^®^ (Tulathromycin; Zoetis Animal Health, Parsippany, NJ), Excede^®^ (Ceftiofur; Zoetis Animal Health, Parsippany, NJ), Micotil^®^ (Tilmicosin; Elanco, Greenfield, IN), Nuflor^®^ (Florfenicol; Merck, Kenilworth, NJ), and Zactran^®^ (Gamithromycin; Boehringer Ingelheim Vetmedica, Duluth, GA). Table 2 displays the cost per milliliter of each antimicrobial from the dataset. The average cost of antimicrobials to dose one 600-pound (272-kg) calf was $15.93, with the highest cost antibiotic being Draxxin (Tulathromycin) and Nuflor (Florfenicol) at $22.73 and the lowest being Excede (Ceftiofur) at $13.56. Table 2 also displays the percentage of metaphylaxis lots that were treated with each antimicrobial. Of the metaphylaxis lots, 39.23% of them were treated using Micotil (Tilmicosin), 23.99% were treated using Draxxin (Tulathromycin), and 23.06% were treated using Zactran (Gamithromycin). Excede (Ceftiofur) and Nuflor (Florfenicol) account for 7.24 and 6.06%, respectively.

**Table 2:** Metaphylaxis treatments used by a U.S. Southern Plains feedlot.

**Trade Name**^a^ **(Drug Name)**	**Price per ml**	**Dosage ml/600 lb**	**Cost to treat 600 lb calf**	**% of Lots Treated**
DRAXXIN (Tulathromycin)	$3.43	6.6	$22.73	23.99%
Excede (Ceftiofur)	$1.51	9.0	$13.56	7.24%
Micotil (Tilmicosin)	$1.37	9.0	$12.37	39.23%
Nuflor (Florfenicol)	$0.63	36.0	$22.73	6.06%
Zactran (Gamithromycin)	$1.30	10.9	$14.17	23.06%
Weighted average			$15.93	

a *DRAXXIN, Zoetis Animal Health, Parsippany, NJ; Excede, Zoetis Animal Health, Parsippany, NJ; Micotil, Elanco, Greenfield, IN; Nuflor, Merck, Kenilworth, NJ; Zactran, Boehringer Ingelheim, Duluth, GA*.

*The prices used are the actual prices paid during the observation period*.

### Treatment effects

The estimated logit model used to estimate the propensity scores is in Table 3. What is important from these results is the direction of the effects on the probability of treatment. Remember that the treatment is metaphylaxis and the control lots received no metaphylaxis. The logit regression has a pseudo *R*-squared of 0.456. The coefficient for placement weight is negative and significant at the one percent level. As the placement weight of a lot increases, the probability of being treated with metaphylaxis decreases. This is consistent with previous literature that finds lighter-weight animals have a higher risk of being diagnosed with BRD. The dummy variable for cattle purchased from an auction market is positive. Auction market cattle have a higher probability of receiving metaphylaxis. This again supports the literature that auction market cattle should receive metaphylaxis treatment as they have a higher risk score of being diagnosed with BRD (7).

**Table 3:** Parameter estimates of the logit model used to predict whether cattle were given metaphylaxis treatment or not.

**Variable**	**Coefficient**	**S.E**.	***P*** **-value**
Intercept	5.579	0.415	<0.001
Placement weight	−0.011	0.001	<0.001
Sale barn	1.337	0.122	<0.001
Mid South	1.973	0.165	<0.001
South	2.881	0.263	<0.001
Border	0.494	0.126	<0.001
West Coast	4.026	0.451	<0.001
North Plains	−0.070	0.254	0.784
Midwest	3.429	0.303	<0.001
Mixed sex lot	1.957	0.352	<0.001
Heifers	1.049	0.107	<0.001
Holstein	−1.819	0.353	<0.001
Spring	−2.106	0.148	<0.001
Summer	−1.922	0.143	<0.001
Fall	0.156	0.115	0.177

*The dependent variable is whether or not the lot received metaphylaxis treatment. The P-values are associated with the null hypothesis that the corresponding parameter was zero*.

All dummy variables for the origin of location are positive and significant at the 1% level, except for the northern plains. The dropped dummy variable is the state where the feedlot is located. Thus, cattle shipped further distances are more likely to be given metaphylaxis.

Dummy variables for the season of the year show that lots placed in the fall have the highest probability of being treated with metaphylaxis. The dropped dummy variable is for the winter season, and the spring and summer variables have negative coefficients. The fall dummy variable has a positive coefficient, matching results reported in previous literature (7).

The estimated treatment effects are in Table 4. The table presents the estimated ATT, ATC, ATE, and the unmatched sample averages. The ATT is the effect of metaphylaxis on lots that received metaphylaxis, and the measure is the differences shown in Table 4. The ATT results show that propensity score matching cannot identify the effect of metaphylaxis treatment. The signs of all estimated ATTs are opposite of what would be expected. Average daily gain has an estimated ATT of −0.15, and if the propensity score matching had been sufficient, it would have been interpreted to mean that metaphylaxis decreased average daily gain by 0.15 pounds for the lots that received metaphylaxis. Further interpretation of the ATT estimates is that metaphylaxis would increase death loss, increase the number of sick head days, and decrease feed efficiency.

**Table 4:** Estimated effects of cattle being given metaphylaxis treatments at feedlot arrival using propensity score matching.

**Variable**	**Sample**	**Metaphylaxis**	**No Metaphylaxis**	**Difference**	**S.E**.	***T*** **-stat**
Average Daily Gain	Unmatched	3.04	3.52	−0.48***	0.015	−32.12
	ATT	3.05	3.20	−0.15***	0.032	−4.88
	ATC	3.51	3.36	0.15**	0.073	–−2.12
	ATE			−0.15***	0.056	−2.77
Respiratory Death Loss	Unmatched	3.02	1.07	1.95***	0.089	21.83
	ATT	3.00	1.79	1.21***	0.253	4.78
	ATC	1.09	2.20	−1.11**	0.503	2.21
	ATE			1.14***	0.387	2.94
Death Loss in First 45 Days	Unmatched	1.94	0.61	1.33***	0.070	18.94
	ATT	1.95	1.03	0.91***	0.208	4.36
	ATC	0.62	1.47	−0.85***	0.329	2.57
	ATE			0.86***	0.257	3.36
Total Death Loss	Unmatched	3.87	1.65	2.22***	0.104	21.42
	ATT	3.82	2.41	1.41***	0.268	5.27
	ATC	1.68	2.98	–−1.30**	0.576	2.27
	ATE			1.33***	0.444	3.00
Post Processing Medicine Cost	Unmatched	4.73	3.28	1.45***	0.147	9.87
	ATT	4.75	5.09	−0.34	0.348	−0.98
	ATC	3.34	4.55	−1.21**	0.477	2.53
	ATE			0.81**	0.381	2.12
Percent Hospital Head Days	Unmatched	1.13	0.60	0.52***	0.048	10.82
	ATT	1.14	0.93	0.20**	0.093	2.18
	ATC	0.61	1.06	−0.45***	0.131	3.39
	ATE			0.38***	0.105	3.66
Feed Efficiency	Unmatched	6.39	6.05	0.34***	0.052	6.68
	ATT	6.40	6.05	0.35***	0.089	3.87
	ATC	6.04	6.87	−0.82**	0.416	1.98
	ATE			0.70**	0.324	2.16

*ATT, average treatment effect on the treated; ATC, average treatment effect on the control; ATE, average treatment effect; Estimated counterfactuals in bold. Two asterisks denote rejection of the null hypothesis of no difference at P < 0.05, and three asterisks denote rejection at P < 0.01*.

We know that these results cannot be the true effect of metaphylaxis treatment. Past experimental research has shown that metaphylaxis has a positive benefit on lots that receive the treatment and the ATT estimates are unreasonable. The propensity score matching method is unable to adequately correct for the endogeneity issues present in the data. The feedlot must have decided to use metaphylaxis based on information about the cattle that is not in the dataset. Propensity score matching can correct for selectivity only with respect to observed data. These results support Larzelere and Cox's arguments that often there is no valid way to correct for endogeneity/selectivity (26). Past research such as Williams et al. considered a case where higher quality cattle were given the treatment, and so even if the propensity score matching did nothing, the expected sign would still be obtained (27).

Table 5 displays the means for the matching variables used in the logit model to estimate the propensity score before and after matching. The table compares the metaphylaxis lots to the created counterfactual from the no metaphylaxis group. Before matching, the means in each group are significantly different from each other. After matching the means, however, a majority of variables are still significantly different. Matching was unable to create a counterfactual from the no metaphylaxis group that closely resembled the metaphylaxis group.

**Table 5:** Means of variables for cattle lots given metaphylaxis lots and matched counterfactuals.

**Variable**		**Metaphylaxis**	**Counterfactual**	***t*** **-stat**	***P*** **-value**
Placement Weight	Unmatched	609.0	732.0	−33.88	<0.000
	Matched	743.0	727.0	6.27	<0.000
Auction Market	Unmatched	0.721	0.596	7.71	<0.000
	Matched	0.529	0.613	−6.89	<0.001
MidSouth	Unmatched	0.189	0.045	16.02	<0.001
	Matched	0.070	0.046	4.15	<0.001
South	Unmatched	0.085	0.014	12.01	<0.001
	Matched	0.030	0.014	4.32	<0.001
Border	Unmatched	0.197	0.285	−5.92	<0.001
	Matched	0.202	0.286	−8.01	<0.001
West Coast	Unmatched	0.052	0.003	11.67	<0.001
	Matched	0.004	0.003	0.21	0.835
Northern Plains	Unmatched	0.035	0.030	0.84	0.400
	Matched	0.008	0.030	−6.50	<0.001
Midwest	Unmatched	0.057	0.008	10.40	<0.001
	Matched	0.015	0.008	2.57	0.010
Mixed Sex	Unmatched	0.056	0.007	10.64	<0.001
	Matched	0.017	0.007	3.79	<0.001
Heifers	Unmatched	0.463	0.180	20.11	<0.001
	Matched	0.282	0.185	9.35	<0.001
Holsteins	Unmatched	0.051	0.024	4.76	<0.001
	Matched	0.010	0.024	−4.62	<0.001
Spring	Unmatched	0.125	0.335	−14.17	<0.001
	Matched	0.302	0.326	−2.00	0.045
Summer	Unmatched	0.140	0.270	−9.18	<0.001
	Matched	0.259	0.268	−0.84	0.398
Fall	Unmatched	0.396	0.196	13.99	<0.001
	Matched	0.174	0.202	−2.86	0.004

*Matching is based on propensity scores*.

Estimates of the ATC provide results that are unreliable also. These results would imply that if lots that did not receive metaphylaxis treatment had instead been treated with metaphylaxis, they would have seen an increase in average daily gain and a decrease in death loss. Again, if you look at the match for the ATC, propensity score matching does not create similar groups.

The treatment effects that have been estimated claim that metaphylaxis would make higher risk animals of BRD worse off, but make lower risk animals better off. This interpretation does not hold from what is known in the literature. These results do lend some credibility to the idea that feedlots are not overusing metaphylaxis treatment. The inability to obtain an adequate match suggests that the feedlot is mainly treating only the truly high-risk lots of cattle.

### Death loss

Dennis et al. used death loss distributions for high-risk cattle that received metaphylaxis and those that did not receive metaphylaxis (18). The death loss distributions for metaphylaxis treatment were estimated using available data for 550-, 700-, and 850-pound (249-, 317-, and 385-kg) weight groups. The data used in this paper are used to estimate similar distributions. These distributions are shown in Table 6.

**Table 6:** Feedlot death loss for cattle given metaphylaxis and not given metaphylaxis.

**Source**		**Metaphylaxis**		**No Metaphylaxis**	
		**Mean**	**SD**	**Mean**	**SD**
Dennis et al. (18)	550 lb	5.0	3.5	12.3	19.6
	700 lb	4.5	1.7	11.0	9.3
	850 lb	2.7	1.3	6.7	7.2
Feedlot Data	550 lb	4.00	4.88	5.48	19.28
	700 lb	3.11	3.30	4.26	13.04
	850 lb	3.99	7.01	5.46	27.69

*For the feedlot data, these are the actual recorded number for those treated with metaphylaxis. The no metaphylaxis column is projected using the efficacy multipliers from Abell et al. (9)*.

## Discussion

Propensity score matching results show that the endogeneity issue is not corrected. A scenario for what would happen if lots that were treated with metaphylaxis did not receive metaphylaxis is unable to be determined using matching methods. What factors other than cattle weight, state of origin, and whether the cattle were from a sale barn might have proven useful to the analysis? Cattle that have been through a 45-day preconditioning program, for example, might not have been given metaphylaxis (27). Cattle being placed in crowded conditions or near other cattle who are already sick might benefit more from metaphylaxis (20, 28).

Even though the methods used do not allow determining the effect of metaphylaxis use, the data can be used to calculate an alternate death loss scenario following the work of Dennis et al. (18). Dennis et al. also used data from actual feedlots and faced a similar endogeneity problem (29). Their solution was to use assumed values and not even attempt propensity score matching.

Dennis and his colleagues estimated the no metaphylaxis alternative death loss distribution based on the work of Abell et al. who report death loss odds ratios for different drug types (9). Dennis et al. calculate a weighted mean and standard deviation odds ratio to reflect the percentage of each antimicrobial that is administered to cattle (18). The percent of cattle administered each antimicrobial comes from a U.S. Department of Agriculture (USDA) report (30). Taking the inverse of the odds ratio yields a normally distributed metaphylaxis efficacy multiplier.

A comparable no metaphylaxis alternative death loss distribution is estimated using the data available in this paper. Abell et al. report the death loss odds ratio using a figure and do not report the actual value (9). The value of the odds ratio used in the calculation is discerned from the figure as close to the true figure as possible. The odds ratio is weighted by antimicrobial as in Dennis et al., but instead of using the USDA percentages, antimicrobial usage percentages from the available feedlot data reported in Table 2 were used (18). This leads to a different metaphylaxis efficacy multiplier that is representative of the feedlot.

The value of the metaphylaxis efficacy multiplier used by Dennis et al. (18) is not reported, but by backward deduction, it is found to be around 2.45 for the mean and 5.5 for the standard deviation (15). The metaphylaxis efficacy multiplier calculated for the feedlot in this paper is 1.37 for the mean and 3.95 for the standard deviation. The metaphylaxis efficacy multiplier found in this paper is lower than Dennis et al. due to the feedlot's use of Zactran (Gamithromycin) (18). Abell et al. found Zactran to have an odds ratio greater than one, which would mean it increases the chance of BRD mortality (9). The USDA percentages used by Dennis et al. showed only 0.1% of cattle were treated with Zactran (18). The data in this paper show that the feedlot treated 23.06% of cattle with Zactran. Thus, the metaphylaxis efficacy multiplier found is weighting the worst performing antimicrobial by a higher percentage than Dennis et al. (18).

From the data, 550-pound (249-kg) placement weight lots that received metaphylaxis had a mean death loss of 4%, 700-pound (317-kg) lots had a mean death loss of 3.11%, and 850-pound (385-kg) lots had a mean death loss of 3.99%. These differ from Dennis et al. that found mean death loss values for lots that received metaphylaxis of 5.0, 4.5, and 2.7% for 550-, 700-, and 850-pound (249-, 317-, and 385-kg) lots, respectively (18). The high death loss for our 850-pound (385-kg) placement weight lots is likely due to factors other than BRD such as pregnancy in heifers (31). Using the metaphylaxis efficacy multiplier, alternative mean death loss values for 550-, 700-, and 850-pound (249-, 317-, and 385-kg) placement weights if they did not receive metaphylaxis are 5.48, 4.26, and 5.46%, respectively. These values are lower than Dennis et al. (18). This is due to the metaphylaxis efficacy multiplier being lower.

Dennis et al. estimated that on average 550-pound (249-kg) placements lose $104.46/head when not treated for metaphylaxis, high-risk 700-pound (317-kg) cattle lose $99.26/head, and high-risk 850-pound (385-kg) cattle lose $63.36/head relative to treated cattle (15). The results here suggest a lower value of metaphylaxis per head due to a lower metaphylaxis efficacy multiplier and a lower effect of using metaphylaxis on death loss than Dennis et al. (18). For the 550-pound (249-kg) placement weights, the data used here gave a 1.48% mean increase in death loss if metaphylaxis is not used while Dennis et al. assumed a 7.3% mean increase in death loss (18).

## Conclusion

This paper attempted to identify the value of metaphylaxis treatment to the feedlot industry using propensity score matching. Propensity score matching is unable to overcome the endogeneity issues present in the data, which means the feedlot based the decision on more than just knowing the cattle weight, state of purchase, and whether or not the cattle were from a sale barn. Even though the effect of metaphylaxis is not identified, the fact that it is unidentifiable supports the argument that feedlots are following a protocol when administering metaphylaxis treatment. This should temper some fear of metaphylaxis treatment being overused and thus increase the amount of antimicrobials given to healthy animals. In the case of the feedlot that provided the data, they are identifying the type of lots at the highest risk of BRD and treating those lots with metaphylaxis.

This paper has provided actual antimicrobial costs that a feedlot pays that are not readily available in the literature. If this paper fully replicated Dennis et al., a lower value of metaphylaxis would be found but is interpretable for only the feedlot from which the data were provided.

## Data availability statement

The datasets presented in this article are not readily available because the datasets are available from the authors subject to approval of the feedlot that provided the data. Requests to access the datasets should be directed to BH at britt.hicks@okstate.edu.

## Ethics statement

Ethical review and approval was not required for the animal study because the data are historical data from a feedlot. They are not from an experiment. Some of the authors have visited the feedlot and confirmed that the cattle were ethically treated. Written informed consent for participation was not obtained from the owners because These are observational data from a feedlot. They are mostly owned by the feedlot, but some custom fed cattle were included. No information is included that could identify any of the individual owners and it would be impractical to obtain such information.

## Author contributions

WM: data organization, economic and econometric analysis, and writing of the original draft. BB: supervision of the project and resources, developed methodology, economic and econometric analysis, writing, and editing. DP: data curation, methodology, and editing. BH: data curation and editing. All authors contributed to the article and approved the submitted version.

## Funding

Financial support was provided by the Oklahoma Agricultural Experiment Station, USDA National Institute of Food and Agriculture, Hatch Project number OKL03170, and the A.J. and Susan Jacques Chair.

## Conflict of interest

The authors declare that the research was conducted in the absence of any commercial or financial relationships that could be construed as a potential conflict of interest.

## Publisher's note

All claims expressed in this article are solely those of the authors and do not necessarily represent those of their affiliated organizations, or those of the publisher, the editors and the reviewers. Any product that may be evaluated in this article, or claim that may be made by its manufacturer, is not guaranteed or endorsed by the publisher.
